# Regulation of soluble fms-like tyrosine kinase-1 production in response to placental ischemia/hypoxia: role of angiotensin II

**DOI:** 10.14814/phy2.12310

**Published:** 2015-02-25

**Authors:** Sydney R Murphy, Kathy Cockrell

**Affiliations:** 1 Department of Pharmacology and Toxicology, University of Mississippi Medical CenterJackson, Mississippi; 2 Physiology and Biophysics, University of Mississippi Medical CenterJackson, Mississippi

**Keywords:** Angiotension II, placental ischemia, preeclampsia, reduced uterine perfusion pressure, sFlt-1

## Abstract

While soluble fms-like tyrosine-1 (sFlt-1) is implicated in the pathogenesis of hypertension during preeclampsia, the mechanisms leading to the enhanced sFlt-1 production remain unclear. A recent report suggests exogenous angiotensin II (ANGII) stimulates sFlt-1 production in pregnant rats, however, the role of endogenous ANGII in mediating the placental production of sFlt-1 in response to placental ischemia remains unknown. Therefore, the purpose of this study was to determine the role of endogenous ANGII in mediating the placental production of sFlt-1 in response to placental ischemia in pregnant Sprague–Dawley rats. To this end we compared sFlt-1 and ANGII levels from placental explants collected from normal pregnant (NP) and Reduced Uterine Perfusion Pressure (RUPP) rats. sFlt-1 (3271 ± 264 vs. 2228 ± 324 pg/mL, *P* < 0.05) and ANGII levels (43.2 ± 2.8 vs. 26.7 ± 1.9 pg/mL, *P* < 0.05) were higher in placental explants from RUPP rats versus NP rats. Administration of Losartan, an angiotensin type 1 (AT_1_) receptor antagonist, (10 mg/day for 5 days) to RUPP rats significantly reduced plasma levels of sFlt-1 (1432 ± 255 pg/mL, *P* < 0.05) when compared with untreated control rats (3431 ± 454 pg/mL). In addition, RUPP-induced hypertension was significantly reduced (113 ± 2 mmHg vs. 139 ± 2 mmHg, *P* < 0.05). In conclusion, placental sFlt-1 and ANGII production are significantly elevated in response to placental ischemia in pregnant rats. In addition, AT_1_ receptor activation, by endogenous ANGII, appears to play an important role in mediating the placental production of sFlt-1 in response to placental ischemia in pregnant rats.

## Introduction

Preeclampsia (PE), or pregnancy-induced hypertension, is postulated to arise from an imbalance of angiogenic factors (Wang et al. 2009). Recent data show that women with PE have increased circulating levels of soluble fms-like tyrosine kinase-1 (sFlt-1) and reduced levels of vascular endothelial growth factor (VEGF) and placental growth factor (P*l*GF) (Maynard et al. 2003; Ahmad and Ahmed 2004; Nagamatsu et al. 2004; Rajakumar et al. 2005, 2009; Makris et al. 2007; Heydarian et al. 2009), which lead to hypertension, proteinuria, and endothelial dysfunction (Roberts et al. 1989; Maynard et al. 2003). While this angiogenic imbalance has been well documented in preeclamptic patients (Maynard et al. 2003; Tsatsaris et al. 2003; Levine et al. 2004; Lam et al. 2005; Wang et al. 2009) the specific mechanisms which lead to the overproduction of sFlt-1 and reductions in circulating VEGF and P*l*GF remain unclear (Karumanchi and Epstein 2007).

One possible mechanism for the enhanced sFlt-1 production seen in PE is through an increase in placental production of angiotensin II (ANGII). Previous data have shown the placenta to house all the components of the renin–angiotensin system (RAS) (Nielsen et al. 2000; Shah 2005; Herse et al. 2007; Irani and Xia 2008). While circulating levels of renin, angiotensinogen, angiotensin II and plasma renin activity (PRA) are elevated during pregnancy, normal pregnant women are resistant to the presser effects of ANGII, and remain normotensive (Brown et al. 1997). However, during PE there is a systemic reduction in PRA, decreased secretion of aldosterone, an increased sensitivity to ANGII, and a reduction in ANG 1–7, the vasodilatory arm of the RAS (Anton et al. 2008). In addition, there is an increase in the placental gene expression of angiotensinogen, angiotensin type 1 (AT_1_) receptor, and secreted ANGII (Anton et al. 2008). Linking ANGII and sFlt-1 production are data from Zhou et al. (2007) demonstrating that exogenous administration of ANGII is a potent stimulus of sFlt-1 production from isolated human placental explants. However, whether increases in endogenously produced ANGII in response to placental ischemia is an important stimulus for the enhanced sFlt-1 production seen in PE remains unclear. Therefore, we hypothesize that endogenous placental ANGII may be an important stimulus for the enhanced sFlt-1 production and resultant hypertension in response to placental ischemia in pregnant rats. Thus, the purpose of this study was to determine the role of endogenous ANGII in mediating the placental production of sFlt-1 and hypertension in response to placental ischemia. In addition, we determined if inhibition of endogenous ANGII with Losartan, an AT_1_ receptor antagonist, could attenuate the elevations in blood pressure and increased sFlt-1 production in response to placental ischemia in pregnant rats.

## Methods and Materials

All studies were preformed in timed-pregnant Sprague–Dawley rats purchased from Harlan, Inc. (Indianapolis, Indiana). Animals were housed in a temperature-controlled room (23°C) with a 12:12 light: dark cycle. All experimental procedures executed in this study were in accordance with National Institutes of Health guidelines for the use and care of animals. All protocols were approved by the Institutional Animal Care and Use Committee (IACUC) at the University of Mississippi Medical Center.

### Model of chronic placental ischemia in pregnant rats (RUPP)

Timed-pregnant Sprague–Dawley rats were randomly assigned to normal pregnant (NP) (*n* = 12) or RUPP (*n* = 12) groups. On day 14 of gestation, animals entering the RUPP group underwent the clipping procedure as described in detail, previously (Granger et al. 2006; Sholook et al. 2007). Control, normal pregnant rats underwent a sham operation. An additional group of normal pregnant rats (*n* = 12) and RUPP (*n* = 12) pregnant rats were orally treated (drinking water) with an AT_1_ receptor antagonist, Losartan (10 mg/day for 5 days) as previously demonstrated (LaMarca et al. 2009). On day 19 of gestation, blood pressure was monitored and blood and tissue samples were collected.

### Measurement of arterial pressure in chronically instrumented, conscious rats

Arterial pressure was determined in all groups of rats on day 19 of gestation. Pregnant rats were catheterized on day 18 under a short-acting anesthetic, isoflurane, delivered by an anesthesia apparatus. A catheter of V-3 tubing (SCI) was inserted into the carotid artery for direct monitoring of blood pressure. The catheter was tunneled to the back of the neck and exteriorized after implantation. On day 19 of gestation, pregnant rats were placed in individual restraining cages for arterial pressure measurements. Arterial pressure was monitored with a pressure transducer (Cobe III Transducer CDX Sema, Birmingham, AL) and was recorded continuously for two 20-min periods after 30 min of stabilization. Rats were then anesthetized for blood and tissue collection.

### Villous explant culture

On day 19 of gestation, the animals were sacrificed and the placentas were collected and washed in ice-cold PBS. The villous explants were then collected using a protocol adapted from Caniggia et al. (2000). The outer membrane was then cut away, the myometrium isolated from the decidua, and the vascular bundles were excised. The villous bundle was plated on 0.4 *μ*m culture plate inserts (Millicell-CM, Millipore Co., Bedford, MA) precoated with 500 *μ*L Matrigel matrix (High concentration (cat#354248), BD Biosciences, San Jose, CA). The tissue was cultured in serum-free DMEM/F12 (ATCC, cat#30-2006) media supplemented with 100 *μ*g/mL streptomyocin, 100 U/mL penicillin, and 0.25 *μ*g/mL ascorbic acid, pH 7.4. Normal pregnant explants were maintained in standard condition at 6% O_2_, 5% CO_2_, 89% N_2_, whereas explants isolated from placenta of RUPP rats were maintained in hypoxic conditions of 1% O_2_, 5% CO_2_, 94% N_2_. Culture media were collected after 3, 22, and 30 h, and were replaced with 1 mL of fresh media. Tissues were collected after 22 h of culture and stored at −80°C.

### Determination of circulating and placental sFlt-1 production

Circulating and placental levels of sFlt-1 were measured by a commercially available ELISA kit (R&D Systems, Quantikine, Minneapolis, MN). Culture media samples were diluted with calibrator diluent (1:2) prior to assay. The assay displayed a sensitivity level of 9.8 pg/mL and interassay variability of <10% and intra-assay of <10%.

### Determination of placental ANGII production

Placental ANGII production was measured by high-performance liquid chromatography (HPLC) at Wake Forest University (Winston-Salem, NC) from NP and placental ischemic (RUPP) pregnant rats after 22 h of culture.

### Statistical analysis

All data are expressed as mean ± standard error of the mean. Differences between control and experimental groups were analyzed using unpaired *t*-tests. Data were considered statistically different at *P* values <0.05. Blood pressure comparisons for multigroup and multifactorial analyses were performed using ANOVA with Student Newman Keul's post hoc test.

## Results

### Placental production of sFlt-1 from villous explants isolated from NP and RUPP rats

**Figure 1:**
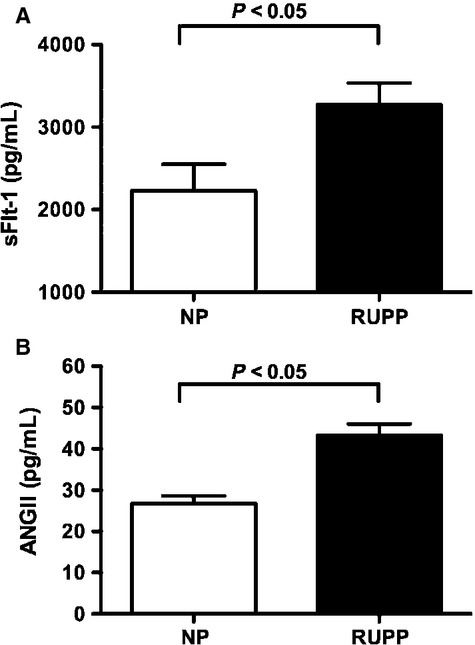
Production of sFlt-1 (Panel A) and ANGII (Panel B) from explants isolated from normal pregnant (NP) and reduced uterine perfusion pressure (RUPP) rats. NP placental explants were cultured for 30 h in normoxic conditions of 6% O_2_, 5% CO_2_, 89% O_2_ whereas RUPP placental explants were maintained at 1% O_2_, 5% CO_2_, 94% O_2_ for 30 h prior to collect of media. Data expressed as mean ± SEM. A *P* value of 0.05 was considered statistically significant.

Consistent with what has been previously reported in women with PE (Ahmad and Ahmed 2004; Rajakumar et al. 2005, 2009; Heydarian et al. 2009) we found placental sFlt-1 production to be significantly elevated in response to placental ischemia in pregnant rats (RUPP, *n* = 12) when compared with NP (*n* = 12) controls, 3271 ± 264 pg/mL versus 2228 ± 324 pg/mL (*P* < 0.05), respectively (Fig.1A).

### Placental production of ANGII from villous explants isolated from NP and RUPP rats

Local production of ANGII within the placenta was significantly elevated in response to placental ischemia (43.2 ± 2.8 pg/mL, *n* = 6) when compared with NP (26.7 ± 1.9 pg/mL, *n* = 5) control (Fig.1B).

### Plasma sFlt-1 levels and arterial pressure responses in NP and RUPP pregnant rats

**Figure 2:**
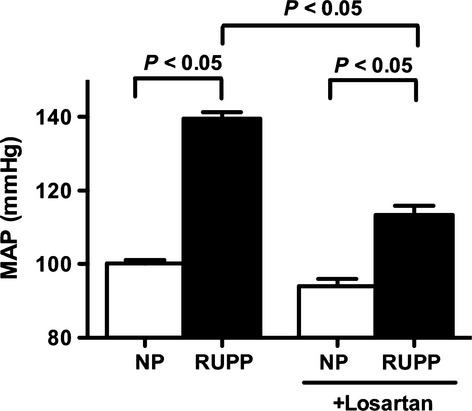
Mean arterial pressure (MAP) in normal pregnant (NP) and reduced uterine perfusion pressure (RUPP) rats treated with and without an AT_1_ receptor antagonist, Losartan (10 mg/day for 5 days in drinking water). MAP was measured in conscious rats on gestational day 18 prior to sacrifice. Data expressed as mean ± SEM. A *P* value of 0.05 was considered statistically significant.

**Figure 3:**
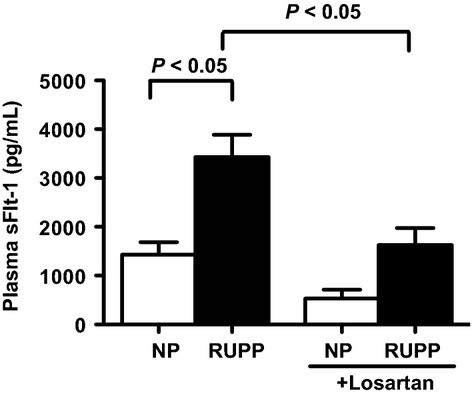
Plasma levels sFlt-1 in normal pregnant (NP) and reduced uterine perfusion pressure (RUPP) rats treated with and without an AT_1_ receptor antagonist, Losartan (10 mg/day for 5 days in drinking water). Data expressed as mean ± SEM. A *P* value of 0.05 was considered statistically significant.

Placental ischemia in pregnant rats significantly increased mean arterial pressure (139 ± 2 mmHg, *n* = 8, *P* < 0.05), compared to NP rats (100 ± 1 mmHg, *n* = 10) (Fig.2). Associated with the hypertension, plasma sFlt-1 concentrations increased approximately threefold when compared with NP control rats (3431 ± 454, *n* = 8 vs. 1432 ± 255 pg/mL, *n* = 10 *P* < 0.05) (Fig.** **3).

### Effect of AT_1_ receptor blockade on plasma sFlt-1 levels in response to reductions in uterine perfusion pressure in pregnant rats

Treatment with Losartan (10 mg/day for 5 days), an AT_1_ receptor antagonist, significantly reduced the hypertension in response to placental ischemia (113 ± 2 mmHg, *n* = 9, *P* < 0.05) while having no effect on normotensive, normal pregnant rats (94 ± 2 mmHg, *n* = 9) (Fig.2). Furthermore, plasma levels of sFlt-1 were significantly decreased in response to AT_1_ receptor antagonism when compared with untreated controls (1626 ± 350 pg/mL, *n* = 9, *P* < 0.05). Losartan treatment did not significantly reduce sFlt-1 concentrations in NP controls (530 ± 182 pg/mL, *n* = 9) (Fig.** **3).

## Discussion

While PE is the leading cause of maternal death during pregnancy, the mechanisms underlying the pathogenesis of the disease remain elusive. Hypertension, proteinuria, and intrauterine growth retardation are believed to result from a lack of spiral artery remodeling of the maternal uterine vasculature supplying the fetoplacental unit (Roberts 2000; Wang et al. 2009). This lack of remodeling is thought to be the result of an increased circulating concentration of anti-angiogenic factors, such as sFlt-1, and subsequent reductions in pro-angiogenic factors, such as VEGF and P*l*GF (Wang et al. 2009). During normal pregnancy, the balance between pro- and anti-angiogenic factors is tightly regulated. However, during PE, this regulation is lost and there is an over production of placental sFlt-1 (Nagamatsu et al. 2004; Nevo et al. 2006; Makris et al. 2007) by mechanisms yet to be fully elucidated.

Involvement of the renin–angiotensin system in the pathogenesis of preeclampsia has been suggested. Recent data demonstrate the placenta houses its own RAS, independent of the renal RAS (Herse et al. 2008; Irani and Xia 2008), and the two may have a reciprocal relationship during PE, such that when the uterine RAS is increased, the renal and systemic RAS are decreased (Shah 2005). Systemic levels of plasma renin concentration, plasma renin activity (PRA), and ANGII levels are elevated during normal pregnancy; but all components of the RAS, are reduced in PE patients, systemically (Skinner et al. 1972; Merrill et al. 2002). In addition, placental levels of ANGII are elevated in PE women along with significant elevations in the presence of the AT_1_ receptor (Nielsen et al. 2000). Thus, the local production of ANGII within the placenta may play an important role in stimulating the over production of sFlt-1 during PE.

While data show a correlation between the placental production of ANGII and sFlt-1 in women with PE, whether ANGII plays a role in stimulating placental sFlt-1 production remains unclear. Therefore, this study investigated whether endogenous ANGII contributes to the enhanced placental production of sFlt-1 and elevations in mean arterial pressure in response to placental ischemia in pregnant rats via AT_1_ receptor activation. The rat is a very useful model for studying preeclampsia because the rat placenta is very similar in anatomy and structure to that of a human (Ain et al. 2006; Konno et al. 2007). In addition, the rat placenta undergoes extensive vascular remodeling and trophoblast cell invasion similar to humans and in contrast to that of the mouse. The RUPP rat is an ideal animal model for the study of mechanisms leading to placental ischemia/preeclampsia and very closely mimics the human syndrome of preeclampsia (Granger et al. 2006). In this report, we found that placental sFlt-1 production was significantly enhanced in placental explants isolated from RUPP rats when compared with normal pregnant placental explants. This correlates with data from human studies in which Nevo et al. (2006) demonstrated a temporal change in placental sFlt-1 mRNA expression throughout gestation and showed placental sFlt-1 mRNA expression to be ten times greater in women with preeclampsia relative to term control and preterm labor patients. Additionally, reports examining animal models of placental ischemia/insufficieny, Makris et al. (2007) showed induction of uteroplacental ischemia in baboons lead to elevated plasma levels of sFlt-1 in addition to an increase in placental sFlt-1 mRNA expression. Furthermore, data from Gilbert et al. (2007) demonstrated sFlt-1 production to be elevated in the placenta of the RUPP rat.

This report shows placental ANGII production is increased from placental ischemic RUPP rats relative to NP rats. Thus, we next determined if ANGII might play a role in mediating the increased production of sFlt-1 and hypertension in response to chronic placental ischemia in pregnant rats. Consistent with what we have previously reported (Gilbert et al. 2007), we found significant elevations in mean arterial pressure in response to placental ischemia in pregnant rats that was associated with an approximate threefold increase in circulating levels of sFlt-1. In addition, treatment with Losartan significantly reduced the hypertension in response to placental ischemia, while decreasing circulating levels of sFlt-1. Therefore, it can be suggested that AT_1_ receptor activation stimulates the placental production of sFlt-1 and contributes to the hypertension in response to placental ischemia.

However, it cannot be discounted that other components of the RAS may contribute to the enhanced sFlt-1 production seen during PE. Recent evidence of an agonistic autoantibody aimed at the AT_1_ receptor (AT1-AA) may be the key player (Wallukat et al. 1999; Dechend et al. 2006; Herse et al. 2008; Karumanchi and Lindheimer 2008). In a study by Zhou et al. (2008), IgG isolated from the serum of PE patients, significantly increased the placental production of sFlt-1 from human placental explants, an effect that was blocked with an AT_1_ receptor antagonist. More recent data from Parrish et al. (2010). achieved a similar response in placental explants from normal pregnant rats incubated in the presence of IgG isolated from serum of RUPP placental ischemic rats. They also demonstrated that infusion of the AT1-AA into NP rats increased circulating sFlt-1. Furthermore, infusion of IgG isolated from the serum of women with PE into NP mice increased circulating sFlt-1 levels, a response that was not seen in nonpregnant mice. Thus, it can be suggested the placenta must be present for the AT1-AA to stimulate sFlt-1 production. However, due to fatal complications in fetal renal development with AT_1_ receptor blockade during pregnancy, alternate autoimmune therapy options warrant further investigation.

## Perspectives

Recent data suggest an imbalance of angiogenic factors during pregnancy may lead to clinical manifestations of the preeclampsia. While previous studies suggest placental ischemia leads to increased placental production of ANGII and sFlt-1, data from this study show endogenous placental ANGII may play an important role to stimulate the anti-angiogenic factor, sFlt-1, in response to placental ischemia in pregnant rats. However, blockade of the renin–angiotensin system during pregnancy is known to have deleterious effects on fetal renal development, thus, further investigations into potential therapies still warrant investigation.

## Conflict of interest

None declared.
